# A non-autonomous Hamiltonian diffeomorphism with roots of all orders

**DOI:** 10.1007/s11784-026-01307-6

**Published:** 2026-05-26

**Authors:** Nicolas Grunder, Baptiste Serraille

**Affiliations:** 1 Institut de Mathématiques, Université de Neuchâtel, Rue Emile-Argand 11, 2000 Neuchätel, Switzerland; 2 Department of Mathematics, ETH-Zürich, Rämistrasse 101, 8092 Zürich, Switzerland

**Keywords:** 37C10 . 37C60 . 37J65 . 53D99 . 57S99

## Abstract

We present a way of constructing non-autonomous Hamiltonian diffeomorphisms with roots of all orders by adapting the Anosov–Katok construction. This answers a question by Kathryn Mann and Egor Shelukhin. Additionally, we construct an action of the rationals by diffeomorphism on any manifold that is not $$ C^0$$-continuous with respect to the Euclidean topology on $$\mathbb {Q}$$.

## Introduction

### Main result and motivation

Let (M,ω) be a symplectic manifold. We say that a Hamiltonian diffeomorphism is *autonomous* when it is the time-1 map of a flow generated by a time-independent Hamiltonian. The set of all autonomous Hamiltonian diffeomorphisms is denoted by $\textrm{Auto}(M,\omega )$. Autonomous Hamiltonian diffeomorphisms enjoy properties that are generally not true for general Hamiltonian diffeomorphisms; for example, if *H* is a time-independent Hamiltonian, then each level set $H^{-1}(\{c\})$ for $c \in \mathbb {R}$ is preserved under the Hamiltonian flow $\varphi ^t_H$. In many ways, the set $\textrm{Auto}(M,\omega )$ is believed to be small in the group of all Hamiltonian diffeomorphisms $\textrm{Ham}(M,\omega )$, as first pointed out by Polterovich and Shelukhin [16]. This is a symplectic take on Palis’ heuristic [14]: “Vector fields generate few diffeomorphisms”. Polterovich and Shelukhin [16], followed by Polterovich, Shelukhin and Stojisavljević [17], Zhang [22], Chor [2], and Khanevsky [9] indeed showed that for a wide class of closed symplectic manifolds, there exist Hamiltonian diffeomorphisms arbitrarily far from elements of $\textrm{Auto}(M,\omega )$ with respect to the Hofer norm. A key observation for the proof of the result of Polterovich and Shelukhin [16] is that all autonomous Hamiltonian diffeomorphisms admit roots of all orders. This comes from the fact that if the time-1 map of the Hamiltonian flow generated by $H :M \rightarrow \mathbb {R}$ is φ, then the time-1 map of the Hamiltonian flow generated by *H*/*k* is a *k*th root of φ. It is thus a natural question to ask whether the only maps that have roots of all orders are autonomous. In [20, Section 3], Shelukhin mentions that there are no known examples of non-autonomous Hamiltonian diffeomorphisms with roots of all orders. The question about their existence has been known in the community, it was asked by V. Stojisavljević in 2015 in private communications and was first asked by Kathryn Mann. In this paper, we show the existence of non-autonomous Hamiltonian diffeomorphisms of general symplectic manifolds that admit roots of all orders. Our main result is the following.

#### Theorem 1

For any symplectic manifold (M,ω) there exists an injective group homomorphism

$$\begin{aligned} \Phi {:}\,\mathbb {Q} \rightarrow \textrm{Ham}(M,\omega ), \end{aligned}$$

such that $$\Phi (\mathbb {Q})\cap \textrm{Auto}(M,\omega ) = \{Id\}.$$

Since $\Phi (1) \in \textrm{Ham}(M,\omega )$ is not autonomous but has roots of all orders, the following corollary is immediate.

#### Corollary 1.1

For any symplectic manifold (M,ω), there exists a Hamiltonian diffeomorphism φ that has roots of all orders but is not autonomous. □

We will see that the images of the constructed group homomorphisms Φ lie in the $C^\infty$-closure of the set of autonomous diffeomorphisms. Naturally, we ask the following question.

#### Question 1

Does there exist a Hamiltonian diffeomorphism that has roots of all orders but does not lie in the $C^\infty$-closure of the set of autonomous diffeomorphisms?

As we will show shortly, the proof of Theorem 1 is a consequence of the following proposition.

#### Proposition 1.2

For any symplectic manifold (M,ω) there exists a group homomorphism

$$\begin{aligned} \Phi {:} \,\mathbb {Q} \rightarrow \textrm{Ham}(M,\omega ), \end{aligned}$$

and a non-empty open set $\mathcal {U}\subset M$ such that for all $\ell \in \mathbb {Z}_{\ge 1}$ there exists a point x∈M with a positive orbit

$$\begin{aligned} \mathcal {O}(\Phi (\ell ),x):=\{x, \Phi (\ell )(x), \Phi (2\ell )(x),\ldots \} \end{aligned}$$

that is dense in $\mathcal {U}$.

This proposition implies Theorem 1, as we show now. The existence of orbits of $\Phi (\ell )$ that are dense in a non-empty open set implies that the $\Phi (\ell )$ are not autonomous.

#### Proof of Theorem 1

Suppose that there exists an autonomous Hamiltonian diffeomorphism $\varphi \in \textrm{Auto}(M,\omega )$, generated by *H* such that there exists a point *x* of *M* with an orbit $\mathcal {O}(\varphi ,x)$ that is dense in an open set $\mathcal {U}\subset M$. Since autonomous Hamiltonians are preserved by the action of their flow, we have that *H* is constant on $\mathcal {O}(\varphi ,x)$. However, since the orbit is dense in *U*, the Hamiltonian $H|_U$ restricted to *U* must be constant. Consequently, φ acts as the identity on *U*, and the orbit $\mathcal {O}(\varphi ,x)$ consists of only one point. This contradicts the density of the orbit in the open set *U*.

After applying this argument to $\Phi (\ell )$ for any $\ell \in \mathbb {Z}_{\ge 1}$ we can conclude that

$$\Phi (\mathbb {Q})\cap \textrm{Auto}(M,\omega )=\{Id\}.$$

Note that Φ is injective since for any integer $\ell \in \mathbb {Z}_{\ge 1}$ the Hamiltonian diffeomorphism $\Phi (\ell )$ has a dense orbit in $\mathcal {U}$ and is, in particular, not the identity. This finishes the proof of Theorem 1 assuming Proposition 1.2. □

As we shall see, the arguments we will use are not specific to the symplectic setting and can be applied to any manifold. Naturally, we present some more general applications of our argument to the set of diffeomorphisms of a compact manifold. To study this group, we will equip it with the $C^0$-topology, which is induced by the $C^0$-metric. This metric is defined on the space of $\textrm{Homeo}(M)$ by first choosing an auxiliary Riemannian metric *g* and then defining

$$\begin{aligned} d_{C^0}(f,g) = \max _{x\in M}d_g(f(x),g(x)). \end{aligned}$$

Similar to before, we show the existence of exotic group homomorphisms from the set of rationals to diffeomorphism groups of manifolds.

#### Theorem 2

For any manifold *M* of dimension greater than or equal to 2 or $S^1$, there exists a group homomorphism

$$\Phi {:}\,\mathbb {Q} \rightarrow \textrm{Diff}^\infty (M)$$

that is not $C^0$-continuous.

The following proposition and Remark 1.4 imply this theorem. Firstly, for manifolds of dimension at least 2, we can make a stronger statement as above.

#### Proposition 1.3

For any manifold *M* of dimension at least 2, there exist non-continuous group homomorphisms

$$\begin{aligned} \Phi {:}\,\mathbb {Q} \rightarrow \textrm{Diff}^\infty _c(M) \end{aligned}$$

that are additionally injective and whose images have no isolated points.

We will prove this result by applying the Anosov–Katok construction similarly to how we will do for the proof of Proposition 1.2. However, the argument in the proof fails for dimension one due to the lack of *k*-transitive diffeomorphisms (that is, diffeomorphisms that send a tuple of *k* isolated points to an arbitrary tuple of *k* isolated points).

#### Remark 1.4

For manifolds *M* that admit a free $S^1$-action by diffeomorphisms that is continuous with respect to $C^0$-topology, one can construct non-continuous group homomorphisms $\mathbb {Q}\rightarrow \textrm{Diff}^\infty (M)$ more directly using that the identity has many *p*^th^ roots for any prime *p*. To be more precise, $\mathbb {Q}$ is $\mathbb {Z}$-generated by 1 and $\{p^{-k}\}_{p,k}$ for all *p* prime and *k* positive integer with the relation that $p \cdot p^{-k}=p^{-k+1}$. A group homomorphism from $\mathbb {Q}$ is uniquely defined only by the values on the generators and the aforementioned relation. The flexibility of the choice of the $p^{\textrm{th}}$ root then allows us to pick roots that are $C^0$ far from the identity. This approach has been inspired by a discussion with Ibrahim Trifa.

#### Remark 1.5

For any symplectic manifold (M,ω) the group homomorphism from Theorem 2 and 1.3 can be assumed to take values in $\textrm{Ham}(M,\omega )$. These results are implied by Proposition 3.1 which is a stronger version of Proposition 1.2 but is formulated for $\textrm{Diff}(M)$. Similarly as in the proof of Proposition 1.2 one can ensure that the constructed homomorphism takes values in $\textrm{Ham}(M,\omega )$.

### Organization of the paper

The paper is organised as follows. In Sect. 2, we recall the Anosov–Katok construction and we prove Proposition 1.2 by adapting the construction to our setting. In Sect. 3, we expand the proof of Proposition 1.2 in order to prove Theorem 2. In Sect. 4, we study the continuity of the map defined in Theorem 1 for two other natural topologies on the group of Hamiltonian diffeomorphisms, namely the γ- and Hofer-topologies.

## Proof of Proposition 1.2

This section is dedicated to the proof of Proposition 1.2. *From now on, we will always assume that M* *is a compact manifold, unless otherwise specified.* We recall first some basic definitions and notations of symplectic geometry. We then describe our setup for the Anosov–Katok construction that differs from the usual one. We finish the proof of Proposition 1.2 in Sects. 2.3 and 2.4.

### Preliminaries of symplectic geometry

A symplectic manifold *M* is a manifold endowed with a symplectic form ω, i.e. a closed non-degenerate 2-form. For any symplectic manifold (M,ω), we denote by $\textrm{Symp}(M,\omega )$ the group of *symplectomorphisms* of *M*. Let $H_t :M \rightarrow \mathbb {R}$ be a time-dependent compactly supported Hamiltonian; one defines the *Hamiltonian vector field *$X_H$ *associated with* *H* to be the unique vector field that verifies the following formula

$$\begin{aligned} \iota _{X_H}\omega =-\textrm{d}H. \end{aligned}$$

The flow of the vector field $X_H$ is called the *Hamiltonian flow* of *H* and is denoted by $\varphi ^t_H$. The time-1 maps of Hamiltonian flows are called *Hamiltonian diffeomorphisms*, and altogether, they form a subgroup of $\textrm{Symp}(M,\omega )$ denoted by $\textrm{Ham}(M,\omega )$.

### Setup of the Anosov–Katok construction

The proof consists in constructing a Hamiltonian diffeomorphism with the Anosov–Katok construction, first introduced in [1]. Their construction uses a Hamiltonian $S^1$-action on a symplectic manifold. We generalise the construction to perform it on symplectic manifolds (M,ω) with a smooth Hamiltonian action $S{:}\,\mathbb {R} \rightarrow (\textrm{Ham}(M,\omega ),\tau _{C^\infty })$ such that there exists an invariant connected open subset U⊂M with compact closure and upon which the restriction of the Hamiltonian action $S(\cdot )|_U$ is 1-periodic and induces a free circle action without fixed points denoted by

$$\begin{aligned} S|_U{:}\,S^1\rightarrow \textrm{Ham}(U,\omega ). \end{aligned}$$

Here, $\tau _{C^\infty }$ denotes the Whitney topology (since we assume that *M* is compact, the weak and strong Whitney topologies agree, and we can say Whitney topology). This topology admits a metric that turns $(\textrm{Diff}(M),\tau _{C^\infty })$ into a complete metric space. From the data of *S*, we will construct a sequence of group homomorphisms $(\Phi _n{:}\,\mathbb {R}\rightarrow \textrm{Ham}(M,\omega ))_n$ such that the following hold.

(i) The images of the group homomorphisms $\Phi _n$ are autonomous flows.
(ii) For any $q\in \mathbb {Q}$ the sequence $(\Phi _n(q))_n$ converges in the $C^\infty$-topology. The limits of all rational points fit together to form a group homomorphism
  $$\begin{aligned} \Phi {:}\,\mathbb {Q}\rightarrow \textrm{Ham}(M,\omega ). \end{aligned}$$
(iii) For every $\ell \in \mathbb {Z}_{\ge 1}$, the diffeomorphisms $\Phi (\ell )$ have a dense orbit in *U*.

The existence of such a group homomorphism $\Phi {:}\,\mathbb {Q}\rightarrow \textrm{Ham}(M,\omega )$ proves Proposition 1.2. In the subsequent two sections, we first show how the sequence $(\Phi _n)_n$ that satisfies (i) and (ii) is constructed and then argue that we can improve the construction, additionally achieving (iii).

Let us assume for now that the above construction can be carried out, then once a suitable group action $S:\mathbb {R}\rightarrow \textrm{Ham}(M,\omega )$ exists, we have proven Proposition 1.2. We now show how to construct a suitable action *S* on every symplectic manifold.

Our construction will take place in a compact ball of $\mathbb {R}^{2n}$ endowed with the standard symplectic form $\omega _0$. Darboux’s theorem allows us to embed this construction in any symplectic manifold. Let $u_0{:}\,\mathbb {R}_{\ge 0} \rightarrow [0,1]$ be a function given by

$$u_0(x)= {\left\{ \begin{array}{ll} 1-x, & \quad \text {for }x\in [0,1]\\ 0,& \quad \text {otherwise. } \end{array}\right. }$$

We now locally smoothen $u_0$ in [0,ε) and $(1-\varepsilon , 1+\varepsilon )$, two small neighbourhoods of 0 and 1 respectively. By doing this, we can obtain a smooth compactly supported function $u{:}\,\mathbb {R}_{\ge 0}\rightarrow [0,1]$ that is locally constant at 0 and coincides with $u_0$ on $(\varepsilon , 1-\varepsilon )$. We now define the radial Hamiltonian on $\mathbb {R}^{2n}$ by

$$\begin{aligned} G(x) = u(|x|). \end{aligned}$$

The flow generated by *G* is a suitable Hamiltonian action $S{:}\,\mathbb {R} \rightarrow \textrm{Ham}(\mathbb {R}^{2n},\omega _0)$. That is, denoting $B_r$ for the open ball of radius *r* centred at the origin, the open set $U = B_{1-\varepsilon }-\overline{B_{\varepsilon }}$ is invariant under *S* and *S* acts on *U* as a free circle action.

### Scheme of the construction

We construct the sequence of autonomous morphisms $\Phi _n$ with the Anosov–Katok method, following the scheme described in [11]. Our construction differs from theirs in that we use a diagonal argument to impose the convergence of a countable number of maps simultaneously. We denote by $d_{C^\infty }$ a distance on $\textrm{Diff}(M)$ that induces the Whitney $C^\infty$-topology and turns it into a complete metric space.

The backbone of this construction is the Hamiltonian action *S* which restricts to a circle action on an invariant set *U*. Then the morphisms $\Phi _n$ are defined as conjugations of the action *S* stretched by the factor $\alpha _n$. In other words, we have

$$\begin{aligned} \Phi _n(\lambda ) = H_nS_{\lambda \alpha _{n+1}}H_n^{-1}, \end{aligned}$$

where $H_n \in \textrm{Ham}(U,\omega )$ is a Hamiltonian diffeomorphism compactly supported in *U* and $\alpha _n\in \mathbb {Q}$. In addition to this, we also demand that when written in irreducible form $\alpha _n=\frac{p_n}{q_n}$ is such that $p_n$ is divisible by *n*!. In what follows, we will need fast convergence of the sequence of rational numbers $\alpha _n$, we describe briefly why the constraint on the numerator is harmless with respect to that.

#### Lemma 2.1

Let *n* be any integer, then the set of fractions that can be written with a numerator that is divisible by *n*! in their irreducible representation is dense in $\mathbb {R}$.

#### Proof

Let ε>0 be a positive number, pick *q* a prime number larger than *n*! and $\frac{2n!}{\varepsilon }$. Then, the set of all irreducible fractions with denominator *q* and numerator divisible by *n*! forms an ε-net inside $\mathbb {R}$. Since we can do this for all ε>0, the result follows. □

The construction will be inductive; we construct the Hamiltonian diffeomorphism $H_n$ such that $H_n:=H_{n-1} \circ h_n$ for some Hamiltonian diffeomorphism $h_n \in \textrm{Ham}(U,\omega )$ that commutes with $S_{1/q_n}$. In this way for every $\lambda \in \mathbb {Q}$ with denominator in $\llbracket 1, n \rrbracket = [1,n]\cap \mathbb {Z}$, the following holds

1 $$\begin{aligned} \Phi _{n-1}(\lambda ) = H_{n-1} S_{\lambda \alpha _{n}}H_{n-1}^{-1} = H_{n} S_{\lambda \alpha _{n}}H_{n}^{-1}. \end{aligned}$$

The identity (1) implies that if we choose $\alpha _{n+1}$ close enough to $\alpha _n$, we can conclude that for all $k \in \llbracket 1, n \rrbracket$, we have

2 $$\begin{aligned} d_{C^\infty }\left( \Phi _n\left( 1/k\right) ,\Phi _{n-1}\left( 1/k\right) \right)&= d_{C^\infty }(H_n S_{\alpha _{n+1}/k}H_n^{-1}, H_{n-1} S_{\alpha _{n}/k}H_{n-1}^{-1})\nonumber \\&= d_{C^\infty }(H_n S_{\alpha _{n+1}/k}H_n^{-1}, H_{n} S_{\alpha _{n}/k}H_{n}^{-1})\nonumber \\&\le \dfrac{1}{2^n}. \end{aligned}$$

Note that for any $k\in \mathbb {Z}_{\ge 1}$ (2) will hold eventually for large enough *n*. This means that for every $\ell \in \mathbb {Z}_{\ge 1}$, the sequence of maps $(\Phi _n(\frac{1}{\ell }))_n$ is a Cauchy sequence and thus converges in $C^\infty$-topology to a diffeomorphism that we denote $\Phi (\frac{1}{\ell })$. This last diffeomorphism lies in $\textrm{Symp}_0(M,\omega )$ since the group $\textrm{Symp}_0(M,\omega )$ is locally contractible for the $C^1$-topology. Given the pointwise convergence of the sequence $(\Phi _n)_n$ on $\mathbb {Q}$ and the fact that each $\Phi _n$ is a group homomorphism, it follows directly that the sequence converges to a map $\Phi :\mathbb {Q} \rightarrow \textrm{Symp}_0(M, \omega )$, which is also a group homomorphism. Since the diffeomorphisms in the image of Φ are $C^\infty$ limits of Hamiltonian diffeomorphisms and the $C^k$-flux conjecture holds for $1\le k\le \infty$ [13], we can conclude that the limit diffeomorphisms lie in $\textrm{Ham}(M,\omega )$, that is, we have constructed

$$\begin{aligned} \Phi {:}\, \mathbb {Q} \rightarrow \textrm{Ham}(M,\omega ). \end{aligned}$$

This construction is very general; now one can demand additional properties for the sequences $(\alpha _n)_n$ and $(H_n)_n$ to achieve specific properties in the limit Φ. For example, as described in [11], by demanding light conditions for $H_n$, the limit diffeomorphisms can be forced to have a dense orbit in *U* and therefore are not autonomous.

Although the condition on $h_n$ might seem very strong, it can be reformulated as follows. For any $n\in \mathbb {Z}_{\ge 1}$, we can consider the quotient $(U/S_{1/q_n},\sigma _n)$ which is a smooth symplectic manifold with a symplectic structure $\sigma _n$ uniquely determined by $\pi _n^*\sigma _n = \omega$ where

$$\pi _n: U \rightarrow U/S_{1/ q_n}$$

is the standard projection induced by ω. Note that $\pi _n$ is a smooth covering. Any Hamiltonian diffeomorphism $g_n\in \textrm{Ham}(U/S_{1/q_n},\sigma _n)$ admits a lift $h_n\in \textrm{Ham}(U,\omega )$ that commutes with $S_{1/q_n}$ and therefore (1) is achieved.

### Achieving dense orbits

The proof that a dense orbit in the limit diffeomorphisms of the Anosov–Katok construction can be achieved as described in [11]. For the convenience of the reader and since we want the limit diffeomorphisms $\Phi (\ell )$ to have dense orbits for all $\ell \in \mathbb {Z}_{\ge 1}$, we describe how this construction can be achieved.

Fix an auxiliary Riemannian metric on *M* and denote the induced distance by *d*. A subset F⊂U is called ε*-dense* in *U* if

$$\begin{aligned} \sup _{x\in U}\inf _{y\in F}d(x,y)<\varepsilon . \end{aligned}$$

Now choose a fixed sequence $(\varepsilon _n)_n$ of real numbers converging to 0. We will choose $g_{n}\in \textrm{Ham}(U/S_{1/q_n},\sigma _n)$ so that $\Phi _n(n!)$ has an orbit $\mathcal {O}(\Phi _n(n!),x_n)$ that is $\varepsilon _n$-dense in *U*. Note that since for $\ell = 1,\ldots ,n$ we have

$$\begin{aligned} \mathcal {O}(\Phi _n(n!),x_n) \subset \mathcal {O}(\Phi _n(\ell ),x_n), \end{aligned}$$

and thus $\Phi _n(\ell )$ has an $\varepsilon _n$-dense orbit in *U*. Fix $\ell \in \mathbb {Z}_{\ge 1}$, since for *n* an integer large enough, the map $\Phi _n(\ell )$ has an $\varepsilon _n$-dense orbit, we obtain as in [11, Section 2.3] that fast convergence of $\Phi _n(\ell )$ implies that $\Phi (\ell )$ has an ε-dense orbit in *U* for any ε>0. Here, we use the compactness of the closure of *U* to conclude that such orbits can be assumed to be finite. Again, as in [11], this implies that $\Phi (\ell )$ has a dense orbit in *U*. This would imply Proposition 1.2.

We still need to show that the sequence $(g_n)_n$ can be chosen such that $\Phi _n(n!)$ has an $\varepsilon _n$-dense orbit. We assume that we already constructed $\alpha _n$ and $H_{n-1}$ such that the map $\Phi _{n-1}((n-1)!)=H_{n-1} S_{\alpha _n(n-1)!}H_{n-1}^{-1}$ has an $\varepsilon _{n-1}$-dense orbit and construct $H_n$ and $\alpha _{n+1}$ such that $\Phi _n(n!)$ has an $\varepsilon _n$-dense orbit. Let again

$$\begin{aligned} \pi _n{:}\,U\rightarrow U':= U/S_{1/q_n} \end{aligned}$$

be the standard projection. Choose a finite set of points $F_1\subset U'$ such that $\pi _n^{-1}(F_1)$ is $\eta _{n}$-dense in *U* where $\eta _{n}$ is chosen such that $H_{n-1}$ maps any ball of diameter $\eta _{n}$ inside a ball of diameter $\varepsilon _{n}$. Note that this is possible since *U* lies in the support of the flow *S*, which is compact. We choose a set $F_2\subset U'$ with the same cardinality as $F_1$, lying in the projection $\pi _n(C)$ of a single orbit *C* of the circle action on *U*. Since the group of Hamiltonian diffeomorphisms is $|F_1|$-transitive (Lemma 2.2), we can pick $g_n\in \textrm{Ham}(U')$ such that $g_n(F_2)=F_1$.

#### Lemma 2.2

For any manifold *M* and any *p*-tuples $(x_1,\ldots ,x_p)$ and $(y_1,\ldots ,y_p)$ where $x_i \ne x_j$ and $y_i\ne y_j$ for i≠j there exists a compactly supported diffeomorphism ψ such that $\psi (x_i) = y_i$ for all *i*. If we assume the existence of a symplectic form on *M*, then ψ can be assumed to be a Hamiltonian diffeomorphism. □

We now define $$h_n$$ as the lift of $$g_n$$ to *U*. The image $$h_n(C)$$ of the orbit *C* is $$\eta _n$$-dense in *U* and thus, $$H_{n-1}(h_n(C))$$ is $$\varepsilon _n$$-dense in *U*. We choose a rational number $$\alpha _{n+1}=\frac{p_{n+1}}{q_{n+1}}$$ with $$p_{n+1}$$ divisible by (n+1)! such that there is a discrete orbit $C^{\prime }\subset C$ under $$S_{\alpha _{n+1}n!}$$ that contains a subset close enough to $$\pi _n^{-1}(F_2)$$ such that $$H_{n-1}(h_n(C'))$$ is $$\varepsilon _n$$-dense in *U*, this is possible if $$\alpha _{n+1}$$ is close enough to $$\alpha _n$$. We define $$H_n:=H_{n-1}\circ h_n$$ and $$\Phi _n=H_nS_{\alpha _{n+1}}H_n^{-1}$$. The set $$H_n(C')$$ is then an $$\varepsilon _n$$-dense orbit of $$\Phi _n(n!)$$. This concludes the proof of Proposition 1.2.

## Proof of Theorem 2

As explained in the introduction, the proof of Theorem 2 follows from Proposition 1.3. This section is devoted to the proof of Proposition 1.3. The construction scheme is the same as in the previous section. In fact, the construction of Φ can be performed analogously without knowledge of a symplectic form, where diffeomorphisms replace Hamiltonian diffeomorphisms.

### Constructing exotic rationals in $\textrm{Diff}(M)$

Starting from a continuous group homomorphism $\mathbb {R}\rightarrow (\textrm{Diff}(M),\tau _{C^\infty })$ we will build an exotic group homomorphism $\Phi {:}\,\mathbb {Q}\rightarrow \textrm{Diff}(M)$ similarly to the previous section. However, we now demand the group homomorphism Φ to fulfill further conditions.

#### Proposition 3.1

For any smooth manifold *M* of dimension greater than or equal to 2, there exists a group homomorphism

$$\begin{aligned} \Phi {:}\,\mathbb {Q}\rightarrow \textrm{Diff}(M) \end{aligned}$$

satisfying the following properties:

(i) There exists a real number c>0 such that for any prime *p* large enough we have $d_{C^0}(Id,\Phi (1/p))>c$.
(ii) There exists a diverging sequence of *natural numbers* $(p_n)_n$ such that $\Phi (1/p_n)$ converges to the identity.
(iii) For any $\ell \in \mathbb {Z}_{\ge 1}$ there exists x∈M such that $\{\Phi (\ell n)x\}_{n\in \mathbb {Z}_{\ge 1}}$ is dense in some open set U⊂M.

Note that (*iii*) automatically implies that Φ is injective. Otherwise, there would be a natural number ℓ such that $\Phi (\ell )= Id$, and therefore there cannot be a dense orbit of $\Phi (\ell )$ in any open set. To obtain Φ, we again apply the Anosov–Katok method to a group homomorphism $S{:}\,\mathbb {R}\rightarrow \textrm{Diff}(M)$ but demand the sequences $(\alpha _n)_{n}$ and $(h_n)_n$ to satisfy additional conditions. Let us first assume that we have a continuous group homomorphism $S{:}\,\mathbb {R} \rightarrow \textrm{Diff}(M)$ and an open set U⊂M where *S* is invariant and so that *S* restricts to a group action $S|_U{:}\,\mathbb {R} \rightarrow \textrm{Diff}(U)$ with a discrete kernel and therefore induces a free circle action by diffeomorphisms, that is, a continuous group homomorphism $S|_U{:}\,S^1\rightarrow \textrm{Diff}(U)$ such that $S|_U(\xi )(x) =x$ for some x∈U implies that ξ=1. We again choose a sequence $(h_n)_n$ of diffeomorphisms of *M* compactly supported in *U* and a sequence of rational numbers $(\alpha _n)_n$ and define

$$\begin{aligned} \Phi _n{:}\,\mathbb {Q}\rightarrow \textrm{Diff}(M),\quad \lambda \mapsto H_n \circ S_{\lambda \alpha _{n+1}} \circ H_n^{-1} \end{aligned}$$

where $H_n = h_1\circ h_2 \circ \ldots \circ h_n$. Like in the previous section, the sequence $(\Phi _n)_n$ will converge to a group homomorphism Φ. For Φ to satisfy condition (*i*) from Proposition 3.1 we choose $(h_n)_n$ and $(\alpha _n)_n$ in such a way that there exists a real number c>0 such that for any prime *p* we have

3 $$\begin{aligned} d_{C^0} (Id,\Phi _{p-1}(1/p))> 2c. \end{aligned}$$

The sequence $(\Phi _n)_n$ of group homomorphisms will again fulfill condition (2) and therefore we have that

$$d_{C^0}(Id,\Phi (1/p)) \ge d_{C^0}(Id,\Phi _{p-1}(1/p))-d_{C^0}(\Phi _{p-1}(1/p),\Phi (1/p))> 2c - \sum _{k \ge p}2^{-k}.$$

Thus, for large enough primes *p* we have that

$$\begin{aligned} d_{C^0}(Id,\Phi (1/p)) > c. \end{aligned}$$

To achieve (*ii*) it is enough to refine the choice of $\alpha _n = {p_n\over q_n}$ such that $p_n|p_{n+1}$ and $q_n$ is chosen to be large enough. Choosing $(\alpha _n)_n$ correctly will imply that the $C^0$-norm of $\Phi _n(1/p_n)$ converges to 0. As will become apparent later, all this can be done simultaneously while also ensuring $\Phi (\ell )$ has a dense orbit in an open set such that Φ also satisfies (*iii*).

Let us first concentrate on (*i*). For this, we choose $(h_n)$ in a particular way where $h_n$ is supported on an open set $U_n$ where

$$\begin{aligned} U_1\subset U_2 \subset U_3 \subset \ldots \subset U \end{aligned}$$

is an increasing sequence of open sets. This gives us control of the behaviour of $H_n = H_{n-1}\circ h_n$ on $U_n-U_{n-1}$ since $H_{n-1}$ is supported on $U_{n-1}$. More concretely, to prove (3), we will use the following lemma.

#### Lemma 3.2

For any open set V⊂M and free circle action *S* on *V* there exists a connected open set U⊂V and a sequence of connected open sets

$$U_1\subset U_2 \subset U_3 \subset \ldots \subset U$$

and a positive number c>0 such that the following is fulfilled.

(i) $\bigcup _n U_n$ is dense in *U*.
(ii) For all *n* large enough, there exists $$x_{n+1},y_{n+1}$$ in the interior of $$ U_{n+1}-U_n$$ such that $$d(x_{n+1},y_{n+1})>c$$.
(iii) The sets $U_n$ are invariant under the circle action *S*.

#### Proof

Let us first consider the case when dim(M)=2. Then, since we assumed the circle action to be free, the open set *V* is diffeomorphic to a disjoint union of cylinders and tori. Let U⊂V be an open cylinder that is invariant under *S* and such that *U* has two disjoint boundary components A∪B that lie in *U*. Note that *A* and *B* are necessarily disjoint orbits of *S*. Consider

$$U_n = \left\{ x\in U| \inf _{t\in S^1}d(S_tx,\partial U)> {1\over n}\right\} $$

which is an exhausting sequence of *U* by open connected subsets which are invariant under the circle action. Now let d(A,B)=2c>0 and note that for *n* large enough, $U_{n+1}-U_n$ has two connected components, each close to a boundary component *A* or *B*. Picking any $x_{n+1},y_{n+1}$ in distinct connected components of $U_{n+1}-U_n$ will ensure that (ii) holds.

If the dimension of *M* is n>2, let U⊂V be a connected component. We choose $x_0, y_0 \in U$ with distinct continuous orbits $C_{x_0},C_{y_0}$ under *S*. Let *X* denote the vector field that induces the circle action. That is

$$X = {\textrm{d}\over \textrm{d}t}|_{t=0}S_t.$$

For any x∈U and ε>0 choose a smoothly embedded (n-1)-dimensional open disk $D_{x,\varepsilon }$ centred at *x* that is transverse to *X* and contained in a ε-ball centred at *x*. For ε small enough, note that for any $z\in D_{x,\varepsilon }$ we have $\{S_tz\}_{t\in S^1}\cap D_{z,\varepsilon } = \{z\}$. In this case, the set $N_{x,\varepsilon } = \bigcup _{t\in S^1}S_tD_{x,\varepsilon }$ is an $S^1$-invariant neighbourhood of the orbit of *x*. Moreover, for any δ>0 there exists ε>0 such that $N_{x,\varepsilon }$ is contained in a δ-neighbourhood of the orbit of *x*. Moreover, for ε small enough, note that $N_{x_0,\varepsilon }$ and $N_{y_0,\varepsilon }$ are disjoint tubular neighbourhoods of $C_{x_0}$ and $C_{y_0}$. Now consider

$$U_n = U-(N_{x_0,\varepsilon /n}\cup N_{y_0,\varepsilon /n})$$

and note that these sets form an exhausting sequence $U-(C_{x_0}\cup C_{y_0})$ of open connected sets invariant under the circle action. Since $U-(C_{x_0}\cup C_{y_0})$ is dense in *U* the sequence fulfills (i). For similar reasons as in the case of dimension 2 they also fulfill (ii) if we define $d(C_{x_0},C_{y_0}) = 2c >0$. □

We now show how to construct $(h_n)_n$ so that $(\Phi _n)_n$ satisfies (3). Namely, we first proceed in the same way as in Section 2.3; however, we require that $h_n$ is supported in the open set $U_n$. Recall that we achieve this by choosing diffeomorphisms of the quotients $U_n /S_{1 / q_n}$ and lifting them to compactly supported diffeomorphisms of *U* that send a discrete finite orbit *C* to an $\eta _n$-dense set. Without loss of generality, we may assume that the points $x_n$ and $y_n$ given by Lemma 3.2 are not contained in *C* or its image. In addition to what we have demanded $h_n$ to fulfill up to now, we further ask that when n+1 is prime, $x_n$ be sent to $x_n$ and $S_{\alpha _{n}/n+1}x_n$ be sent to $y_n$,

$$\begin{aligned} h_n(x_n) = x_n,\quad h_n(S_{\alpha _{n}/n+1}(x_n)) = y_n. \end{aligned}$$

In general, this is not possible since $h_n$ is a lift of a diffeomorphism of the quotient $U_n /S_{1 / q_n}$ where we might identify $x_n$ with $S_{\alpha _n/n+1}(x_n)$. This is the case if and only if $\alpha _n = {p_n\over q_n}$ is such that n+1 divides $p_n$. However, if n+1 is prime, we will show in a moment that this is not an issue. As we will see, the described choice of $(h_n)_n$ together with fast convergence of $(\alpha _n)_n$ implies that Φ satisfies conditions (*i*) and (*iii*) from Proposition 3.1. Given the goal of also achieving (*ii*) simultaneously, we will ask that $p_n$ and $q_n$ fulfil even further conditions. Namely, what we need for (*ii*) is that $p_n$ divides $p_{n+1}$. We show that this and all of the above conditions can be achieved by a set of rational numbers that are dense in $\mathbb {Q}$, ensuring that the sequence $(\alpha _n)_n$ can converge arbitrarily fast. Let $\mathcal {Z}_n$ be the set of natural numbers that are given by a product of numbers 1,…,n

$$ \mathcal {Z}_n = \{2^{\ell _1}\cdot \cdots \cdot n^{\ell _{n-1}}|\ell _i \in \mathbb {Z}_{\ge 0}\}. $$

Note that by demanding that $p_n\in \mathcal {Z}_n$ we automatically have that n+1 does not divide $p_n$ if n+1 is prime.

#### Lemma 3.3

Let $n\in \mathbb {Z}_{\ge 2}$ and $p_{n-1}\in \mathcal {Z}_{n-1}$. Then the set of rational numbers ${p_n\over q_n}$ in irreducible form, where $p_n$ belongs to $\mathcal {Z}_n$ is and divisible by *n*! and $p_{n-1}$, is dense in $\mathbb {Q}$.

#### Proof

Let $\frac{p}{q}$ be a rational number and let ε>0. For any choice of $$p_n$$ we first set $$\tilde{q}_n = \lfloor {p_nq\over p}\rfloor $$. At the end of the proof, we will let $$p_n$$ and thus $$\tilde{q}_n$$ run to infinity. We now use the following Sub-lemma.

#### Sub-lemma 3.3.1

Let $r_1, \ldots , r_k$ be natural numbers. Then, there exists some natural number $P{:}{=}P(r_1, \ldots , r_k)$ such that for all $x \in \mathbb {N}$ there is a natural number in the interval $\llbracket x-P, x+P \rrbracket$ that is co-prime with all $r_i$’s.

#### Proof

Let $P=r_1 \cdot \cdots \cdot r_k$, then in each interval of the form $\llbracket x-P, x+P \rrbracket$ there is at least one number *y* that verifies that $y \equiv 1 \pmod {P}$. This implies that this number is not divisible by any of the $r_i$’s. □

From the Sub-lemma, we define $P {:}{=}P(n!,p_{n-1})$ thus there exists $q_n\in \llbracket \tilde{q}_n-P,\tilde{q}_n +P\rrbracket$ that is not divisible by any prime smaller than *n* nor any prime dividing $p_{n-1}$. Thus, $p_n\over q_n$ in irreducible form is given by $\bar{p}_n\over \bar{q}_n$ where $p_{n-1}$ and *n*! still divide $\bar{p}_n$. We then have that

$$ \left| {p_n\over q_n}-{p\over q}\right| = {|p_nq-pq_n|\over q_nq} \le {|p_nq-p\tilde{q}_n|\over q_n q} + {Pp\over q_nq } \le {(P+1)p\over q_n q}. $$

Note that $p_n\in \mathcal {Z}_n$ satisfying $n!|p_n$ and $p_{n-1}|p_n$ can be chosen arbitrarily large. By choosing $p_n$ large enough, we achieve ${(P+1)p\over q_n q}< \varepsilon$ and thus

$$\begin{aligned} \left| {p_n\over q_n}-{p\over q}\right| <\varepsilon . \end{aligned}$$

□

Thus, we can assume that the sequence $(\alpha _n)_n$ is chosen such that $\alpha _n = {p_n\over q_n}$ in its irreducible form is such that *n*! and $p_{\ell }$ divide $p_n$ for any $\ell \le n$ and additionally, if n+1 is prime, n+1 does not divide $p_n$. Moreover, we can choose $\alpha _{n+1}$ arbitrarily close to $\alpha _n$ that satisfies all the required properties discussed in the previous sections and fulfils the following additional constraints. Firstly, we demand $\alpha _{n+1}$ to be so close to $\alpha _n$ such that $h_n\circ S_{\alpha _{n+1}/n+1}(x_n)$ lies in the interior of $U_n\setminus U_{n-1}$. Now consider the map $\Phi _n(1/ n+1) = H_{n-1}h_n S_{\alpha _{n+1}/n+1}h_n^{-1} H_{n-1}^{-1}$ and note that

$$\begin{aligned} \Phi _n\left( 1 \over n+1\right) (x_n)&= H_{n-1} h_n S_{\alpha _{n+1}/n+1}h_n^{-1} H_{n-1}^{-1}(x_n) \\  &= H_{n-1}h_n S_{\alpha _{n+1}/n+1}h_n^{-1}(x_n)\\  &= H_{n-1}(h_n S_{\alpha _{n+1}/n+1}(x_n)) \\  &= h_n S_{\alpha _{n+1}/n+1}(x_n). \end{aligned}$$

The second and last equalities come from the fact that $x_n$ and $h_n S_{\alpha _{n+1}/n+1}(x_n)$ lie outside the support of $H_{n-1}$. We choose $\alpha _{n+1}$ possibly even closer to $\alpha _n$ such that

$$d(y_n, h_n S_{\alpha _{n+1}/n+1}x_n) < d(x_n,y_n)-2c$$

and therefore ensuring that

$$\begin{aligned} d(x_n,\Phi _n(1/ n+1)x_n)>2c. \end{aligned}$$

Thus, we can conclude that

$$\begin{aligned} d(Id,\Phi _n(1/n+1))>2c \end{aligned}$$

and therefore Φ satisfies condition (*i*). To see that condition (*ii*) is fulfilled, first note that for any k≤n we have

$$\begin{aligned} \Phi _k(1/p_n) {=} H_k S_{\ell _{k+1}/ q_{k+1}}H_k^{-1} = H_{k+1}S_{\ell _{k+1}/q_{k+1}}H_{k+1}^{-1} =H_{k+1}S_{\alpha _{k+1}/p_n}H_{k+1}^{-1}, \end{aligned}$$

where $\ell _{k+1} = p_{k+1}/p_n$. By fast convergence of the sequence $(\alpha _n)_n$ we can assume

$$\begin{aligned} d_{C^\infty }(\Phi _k(1/p_n),\Phi _{k+1}(1/p_n))&= d_{C^\infty }(H_{k+1}S_{\alpha _{k+1}/p_n}H_{k+1}^{-1},H_{k+1}S_{\alpha _{k+2}/p_n}H_{k+1}^{-1})\\&\le 2^{-k-1}. \end{aligned}$$

Therefore, we have that

$$\begin{aligned} d_{C^\infty }(\Phi _n(1/p_n),\Phi (1/p_n))\le \sum _{k> n}2^{-k}. \end{aligned}$$

Since we can assume that $q_n$ is arbitrarily large we may assume that

$$d_{C^\infty }(Id, \Phi _n(1/p_n)) = d_{C^\infty }(Id,H_nS_{1/q_n}H_n^{-1})\le 2^{-n}.$$

Thus, we have that

$$\begin{aligned} d_{C^\infty }(Id,\Phi (1/p_n)) \le \sum _{k\ge n} 2^{-k} \end{aligned}$$

which proves that Φ satisfies condition (*ii*). Note that (*iii*) is fulfilled for the same reasons as in the previous section.

To complete our argument, we now give an example of an action $S{:}\,\mathbb {R}\rightarrow M$ that restricts to a free $S^1$ action on an invariant subset. Consider any smoothly embedded thickened loop $\gamma :S^1\times D\rightarrow M$ where *D* is the (n-1)-dimensional open disk. Consider the vector field *X* on $\gamma (S^1\times D)$ that is given by

$$\begin{aligned} X(t,x) = {\textrm{d}\over \textrm{d}t}\gamma (t,x). \end{aligned}$$

Now extend *X* to a smooth vector field on *M* and let *S* be the flow of *X*, which induces an $S^1$-action on $\gamma (S^1\times D)$.

## Relation with the γ-topology and Hofer-topology

In the proof of Theorem 2, we saw that the group homomorphism $\Phi :\mathbb {Q} \rightarrow \textrm{Ham}(M,\omega )$ need not be $C^0$-continuous. In this section, we study the continuity properties with respect to other metrics on $\textrm{Ham}(M,\omega )$. When the map Φ is continuous for some topology, then it extends to a continuous group morphism from the real line to some completion of $\textrm{Ham}(M,\omega )$. For the $C^0$-topology we proved earlier that this does not always happen. However, the answer to this question is different when we ask for the Hofer- or γ-topology on $\textrm{Ham}(\mathbb {R}^{2n},\omega _0)$. We recall some properties of the γ-norm and the Hofer norm. The *Hofer norm* is defined in the following way. For all Hamiltonian diffeomorphisms $\varphi \in \textrm{Ham}(M,\omega ),$

$$ d_H(Id,\varphi ):=\inf \int _0^1 \max _M H_t - \min _M H_t \textrm{d}t. $$

As the notation already indicates, the Hofer norm is induced by a metric $d_H$ called the *Hofer metric*. One can see that the Hofer metric is indeed a bi-invariant metric. The fact that it is non-degenerate is a deep result proven by Hofer [5] in the case of $\mathbb {R}^{2n}$, Polterovich [15] in the case of rational symplectic manifolds tame at infinity, and Lalonde and McDuff [10] in full generality.

The γ-norm was first defined by Viterbo in [21] using the theory of generating functions for Lagrangians in the cotangent bundle of a manifold, and it satisfies the following properties. We recall that, as defined in Sect. 2.1, the group $\textrm{Ham}(\mathbb {R}^{2n})$ denotes the set of all *compactly supported* Hamiltonian diffeomorphisms. The construction has been later extended by Schwarz [18] for symplectically aspherical manifolds and by Oh [12] for closed symplectic manifolds.

### Proposition 4.1

(Viterbo). There exists a map $\gamma :\textrm{Ham}(\mathbb {R}^{2n},\omega _0) \rightarrow \mathbb {R}$ that verifies the following properties for all $\varphi , \psi \in \textrm{Ham}(\mathbb {R}^{2n})$.

(i) $$\gamma (\varphi \psi )\le \gamma (\varphi )+\gamma (\psi )$$.
(ii) $\gamma (\varphi )\ge 0$ and $\gamma (\varphi )=0$ if and only if φ=Id.
(iii) $$\gamma (\psi \varphi \psi ^{-1})=\gamma (\varphi )$$.
(iv) $$\gamma (\varphi ) \le d_H(\varphi ,Id)$$.
(v) If $0 \le H \le K$ are two Hamiltonians, then $\gamma (\varphi ^1_H) \le \gamma (\varphi ^1_K)$.

The normed space $(\textrm{Ham}(\mathbb {R}^{2n},\omega _0),\gamma )$ is not complete, and we denote $\widehat{\textrm{Ham}}(\mathbb {R}^{2n},\omega _0)$ its completion called the *Humilière-completion*, see [6]. Note that elements of $\widehat{\textrm{Ham}}(\mathbb {R}^{2n},\omega _0)$ need not be maps. It turns out that Φ as in Theorem 1 constructed above is both γ- and Hofer-continuous and hence can be extended over the completion; this is the content of the next proposition. Propositions 4.2 and 4.3 are formulated for the γ-norm, but they can also be formulated in terms of the Hofer norm. This choice was made because the completion of $\textrm{Ham}(M,\omega )$ for the Hofer-norm is not studied as much as the Humilière-completion.

### Proposition 4.2

The map

$$\begin{aligned} \Phi {:}\,\mathbb {Q} \rightarrow \textrm{Ham}(\mathbb {R}^{2n},\omega _0) \end{aligned}$$

as constructed above is γ-continuous. As a consequence, Φ extends as a homomorphism in the Humilière-completion

$$\begin{aligned} \widehat{\Phi }{:}\,\mathbb {R} \rightarrow \widehat{\textrm{Ham}}(\mathbb {R}^{2n},\omega _0). \end{aligned}$$

Moreover, the map $\widehat{\Phi }$ is injective.

### Proof

Since Φ is a group homomorphism, it suffices to show that Φ is γ-continuous at 0.

Let $\Phi _n(t)=H_n S_{\alpha _n t}H_n^{-1}$ be the sequence of maps constructed in Sect. 2 and let *r* be a rational number. By construction, we know that the sequence of maps $\Phi _n(r)$ converges in the $C^\infty$-topology to Φ(r), hence also in the γ-topology. This and the conjugation-invariance of the γ-norm implies that

$$\begin{aligned} \gamma (\Phi (r))&=\lim _{n \rightarrow \infty } \gamma (\Phi _n(r))\\&=\lim _{n \rightarrow \infty } \gamma (H_nS_{\alpha _n r}H_n^{-1})\\&=\lim _{n\rightarrow \infty } \gamma (S_{\alpha _n r})\\&=\gamma (S_{\alpha r}) \end{aligned}$$

where $\alpha \in \mathbb {R}$ is the limit of the sequence $(\alpha _n)_n$. Therefore, we find $\lim _{r \rightarrow 0}\gamma (\Phi (r))=0$ as expected. Thus, we can define $\widehat{\Phi }:\mathbb {R}\rightarrow \widehat{\textrm{Ham}}(\mathbb {R}^{2n},\omega _0)$ the extension of Φ to the real line. To show that the map is injective, we need to notice that for all $t \in \mathbb {R}$, $\gamma (\widehat{\Phi }(t))=\gamma (S_{\alpha t})$ vanishes if and only if $S_{\alpha t}=Id$ that is if and only if t=0. □

### Proposition 4.3

The image of the extension $\widehat{\Phi }$ of Proposition 4.2 is closed for the γ-topology inside $\widehat{\textrm{Ham}}(\mathbb {R}^{2n},\omega _0)$.

### Proof

Let $\varphi \in \widehat{\textrm{Ham}}(\mathbb {R}^{2n},\omega _0)$ be a point in the γ-closure of $\text {Im}(\widehat{\Phi })$. We show that φ is in $\text {Im}(\widehat{\Phi })$. Since $\widehat{\textrm{Ham}}(\mathbb {R}^{2n},\omega )$ is a metric space, there exists a sequence $x_n \in \mathbb {R}$ such that the sequence $(\widehat{\Phi }(x_n))_n$ γ-converges to φ. Hence, the sequence $(\widehat{\Phi }(x_n))_n$ is a Cauchy sequence and therefore

$$ \gamma (\widehat{\Phi }(x_n) (\widehat{\Phi }(x_m))^{-1}) \xrightarrow [n,m \rightarrow +\infty ]  0. $$

Using the fact that we have a group homomorphism, we get

$$\begin{aligned} \gamma (\widehat{\Phi }(x_n-x_m))\xrightarrow [n,m \rightarrow +\infty ] 0. \end{aligned}$$

However, as shown above, $\gamma (\widehat{\Phi }(x_n-x_m))=\gamma (S_{\alpha (x_n-x_m)})$. Since *S* is generated by a positive Hamiltonian in our construction (see the beginning of Sect. 2), we deduce from Proposition 4.6 and Corollary 4.8 in [21] that the map $t \mapsto \gamma (S_{\alpha t})$ increases on $\mathbb {R}_{\ge 0}$. This implies that

$$\begin{aligned} x_n-x_m \xrightarrow [n,m \rightarrow +\infty ]  0, \end{aligned}$$

in other words the sequence $(x_n)_n$ is a Cauchy sequence itself. In particular, $(x_n)_n$ converges to $x \in \mathbb {R}$ and by γ-continuity,

$$\begin{aligned} \widehat{\Phi }(x_n) \xrightarrow [n\rightarrow +\infty ]{\gamma } \widehat{\Phi }(x)=\varphi \in \widehat{\textrm{Ham}}(\mathbb {R}^{2n},\omega _0). \end{aligned}$$

Thus, φ is indeed in $\text {Im}(\widehat{\Phi })$. □

### Remark 4.4

Combining Proposition 4.2 and Proposition 3.1, item (*i*), we have constructed Hamiltonian diffeomorphisms that have arbitrarily small γ-norm while they have large $C^0$-norm.

We ask the following natural question.

### Question 2

Can we associate elements of $\text {Im}(\widehat{\Phi })$ with honest maps? Are they homeomorphisms? Are they diffeomorphisms?

### The special case of manifolds with a Hamiltonian circle action

In this section, we restrict our attention to the case where the symplectic manifold (M,ω) admits a Hamiltonian circle action $S :S^1 \rightarrow \textrm{Ham}(M,\omega )$. In this setting, we can adapt Proposition 4.1 and Proposition 4.2 to prove the following. Note that the γ-norm can be defined on every symplectic manifold using Floer homology by a work of Fukaya and Ono [3]. In general, the γ-norm enjoys the same properties as the one defined by Viterbo, except for property (v) of Proposition 4.1.

#### Proposition 4.5

Let (M,ω) be a symplectic manifold with a Hamiltonian circle action *S*. Then, the map

$$\begin{aligned} \Phi :\mathbb {Q} \rightarrow \textrm{Ham}(M,\omega ) \end{aligned}$$

as constructed in Theorem 1 is γ-continuous. As a consequence, Φ extends as a homomorphism in the Humilière-completion

$$\begin{aligned} \widehat{\Phi } {:}\,\mathbb {R} \rightarrow \widehat{\textrm{Ham}}(M,\omega ). \end{aligned}$$

Moreover, the map $\widehat{\Phi }$ is 1/α-periodic for an irrational number $\alpha \in \mathbb {R}$ and descends to a map, still denoted $\widehat{\Phi }$,

$$\begin{aligned} \widehat{\Phi } :S^1\rightarrow \widehat{\textrm{Ham}}(M,\omega ). \end{aligned}$$

This last map is injective and its image is closed for the γ-topology.

#### Proof

As in the proof of Proposition 4.2, we have that

$$\begin{aligned} \gamma (\Phi (r))=\gamma (S_{\alpha r}). \end{aligned}$$

Since the map $t \mapsto S_{\alpha t}$ is 1/α-periodic and $\widehat{\Phi }$ is a group homomorphism, we can conclude that $\widehat{\Phi }$ is also 1/α-periodic by the non-degeneracy of the γ-norm.

To show that the image is closed, we adapt the proof of Proposition 4.3 by noticing that the map $t \mapsto \gamma (S_{\alpha t})$ is 1/α-periodic, continuous and takes the value 0 only at integer multiples of $\frac{1}{\alpha }$. Hence, a sequence of maps $(\widehat{\Phi }(x_n))_n$ being a Cauchy sequence for the γ-topology implies that the sequence $(x_n)_n$ is also Cauchy in $S^1$, we conclude the proof as in Proposition 4.3. □

We are still unable to answer Question 2 in this setting, but we have the following partial answer for $(\mathbb {C}P^n,\omega _{FS})$ the standard symplectic projective space. We recall that in this case, the γ-norm is known to be continuous for the $C^0$-topology; Shelukhin [19] proved this in 2018 and Kawamoto [8] gave another proof afterwards.

#### Proposition 4.6

Let

$$\begin{aligned} \widehat{\Phi } :S^1\rightarrow \widehat{\textrm{Ham}}(\mathbb {C}P^n,\omega _{FS}) \end{aligned}$$

be the extension defined in Proposition 4.5 and assume that the rotation number of Φ(1) is exponentially Liouville. Then there exists an uncountable subgroup $G \subset S^1$ such that all the elements in $\widehat{\Phi }(G)$ are homeomorphisms.

In the construction of the map Φ of Theorem 1, we say that the number $\alpha \in \mathbb {R}$ that is the limit of the sequence $(\alpha _n)$ is the *rotation number* of the Hamiltonian diffeomorphism Φ(1). We also say that a real number α is *exponentially Liouville* if

$$ \forall c >0, \exists k,n \in \mathbb {Z}\text { s.t. } 0< \vert k\alpha -n\vert < e^{-ck}. $$

The set of exponentially Liouville numbers is residual and hence non-empty; see [4].

To prove Proposition 4.6, we need the following two lemmas and one corollary. We postpone their proof to the end of this section.

#### Lemma 4.7

A complete metric space with no isolated points is uncountable.

Joksimovic and Seyfaddini have proved the following theorem in [7]. We recall first that a map φ is said to be $C^0$*-rigid* if there exists a sequence of positive integers $n_k$ such that

$$ \varphi ^{n_k} \xrightarrow [k\rightarrow +\infty ]{C^0}Id. $$

#### Theorem A

(Joksimovic-Seyfaddini [7]). Let (M,ω) be a symplectic manifold with a Hamiltonian circle action *S* and Φ the map constructed above. Assume that the rotation number of Φ(1) is exponentially Liouville. Then, the map Φ(1) is $C^0$-rigid.

This Theorem has the following implication.

#### Corollary 4.8

Under the same assumption as in Theorem A, the set $\text {Im}(\Phi )\subset \textrm{Ham}(M,\omega )$ has no isolated point for the $C^0$-topology.

#### Proof of Corollary 4.8

From Theorem A, we get $(n_k)_k$ a sequence of integers such that for the $C^0$-topology $\lim _{k\rightarrow \infty }\Phi (n_k)=0$. This implies that Id=Φ(0) is not isolated, since Φ is a group homomorphism. Then for every $q \in \mathbb {Q}$, the point Φ(q) is also not isolated. This finishes the proof of the corollary. □

We can now prove Proposition 4.6.

#### Proof of Proposition 4.6

Since $\text {Im}(\Phi )$ is an abelian subgroup of $\textrm{Ham}(\mathbb {C}P^n,\omega _{FS})$ and using the $C^0$-continuity of the γ-norm, its closure for the $C^0$-topology is also an abelian subgroup of $\widehat{\textrm{Ham}}(\mathbb {C}P^n,\omega _{FS})$. We have the following inclusions

$$ \overline{\text {Im}(\Phi )}^{C^0}\subset \text {Im}(\widehat{\Phi }) \subset \widehat{\textrm{Ham}}(\mathbb {C}P^n,\omega _{FS}). $$

From Proposition 4.5, we get that

$$ G:=\widehat{\Phi }^{-1}\left( \overline{\text {Im}(\Phi )}^{C^0}\right) $$

is a subgroup of $S^1$. From Corollary 4.8, we get that the $C^0$-closure of $\text {Im}(\Phi )$ has no isolated points, and by Lemma 4.7 it is uncountable. This means that *G* is also uncountable, and since its image under $\widehat{\Phi }$ lies in $\overline{\textrm{Ham}}^{C^0}(\mathbb {C}P^n,\omega _{FS})$, the elements of the image are true homeomorphisms. □

If one can show the $C^\infty$-rigidity of maps obtained by the Anosov–Katok method with exponentially Liouville rotation number, one could extend Proposition 4.6 to additionally find an uncountable number of diffeomorphisms in $\text {Im}(\widehat{\Phi })$. We now prove Lemma 4.7.

#### Proof of Lemma 4.7

Let (*X*, *d*) be a complete metric space with no isolated points. We construct $2^\omega$ Cauchy sequences that converge to pairwise different points. We inductively construct sets $A_n$ of $2^n$ pairwise disjoint balls as follows. Let *B* be a ball of radius ε. Since no isolated points exist, we find $B_1,B_2\subset B$ two non-empty disjoint balls with a radius smaller than $\varepsilon \over 2$. We define

$$\begin{aligned} A_{n+1} = \bigcup _{B \in A_n}\{B_1,B_2\} \end{aligned}$$

while $A_0 =\{B\}$ is a singleton of an arbitrary ball. For any decreasing sequence

$$\begin{aligned} E_0\supset E_2\supset \cdots \supset E_n\supset \cdots \end{aligned}$$

of balls in $A = \bigcup _{n\in \mathbb {Z}_{\ge 0}}$ we have that $E =\bigcap _{n\in \mathbb {Z}_{\ge 0}}E_n$ is non-empty (since *X* is complete). There are $2^\omega$ such sequences, which all lead to pairwise disjoint sets *E*. This finished the proof. □

## Data Availability

No datasets were generated or analysed during the current study.
